# Combination of PD-1 inhibitor and chemotherapy for subcutaneous soft-tissue metastasis in postoperative non-small cell lung cancer: A case report

**DOI:** 10.1097/MD.0000000000049957

**Published:** 2026-08-14

**Authors:** Qiang Zeng, Bin Yang, Yang Hu, Yuan Zeng

**Affiliations:** a Department of Intensive Care Unit, Chengdu Shuangliu Hospital of Traditional Chinese Medicine, Chengdu, Sichuan, China; b Department of Oncology, Hubei Cancer Hospital, TongJi Medical College, Huazhong University of Science and Technology, Wuhan, Hubei, China.

**Keywords:** advanced non-small cell lung cancer, case report, chemotherapy, PD-1 inhibitors, subcutaneous soft-tissue metastasis

## Abstract

**Rationale:**

Despite available therapies, patients with advanced non-small cell lung cancer have a poor prognosis, highlighting the need for novel treatment strategies. The combination of immune checkpoint inhibitors and chemotherapy represents a promising approach. This case report describes the efficacy and safety of this combination in a patient with aggressive metastatic disease.

**Patient concerns:**

A 60-year-old male patient, who had undergone surgical resection for primary lung adenocarcinoma, presented with newly developed subcutaneous nodules and symptoms suggestive of adrenal involvement during routine follow-up.

**Diagnoses:**

Histopathological and imaging examinations confirmed recurrent lung adenocarcinoma with metastases to the subcutaneous soft tissues and the adrenal gland.

**Interventions:**

The patient received platinum-based doublet chemotherapy combined with pembrolizumab (an anti-programmed cell death protein 1inhibitor) as second-line systemic therapy.

**Outcomes:**

Following several treatment cycles, imaging revealed marked regression of metastatic lesions, with only minimal treatment-related adverse events reported. The patient achieved a durable clinical response.

**Lessons:**

This case suggests that combining chemotherapy with programmed cell death protein 1 inhibition may provide rapid and effective tumor control in advanced non-small cell lung cancer patients with bulky, symptomatic metastases, while maintaining a manageable safety profile. Further clinical trials are warranted to validate this approach.

## 1. Introduction

Lung cancer is the leading cause of cancer-related deaths globally. Non-small cell lung cancer (NSCLC) accounts for around 85% of lung cancer cases, with over 50% of patients already having metastases at the time of diagnosis.^[1]^ Common sites of metastasis include the liver, bone, brain, and adrenal glands. Subcutaneous soft-tissue metastasis, referring to tumor spread to subcutaneous fat and muscle tissue, is a rare occurrence.^[2,3]^ This type of metastasis typically signifies advanced or end-stage disease.^[4,5]^ When subcutaneous soft-tissue metastasis occurs, it is often in the context of widespread metastases throughout the body, resulting in a significant tumor burden.

Immunotherapy has emerged as a promising approach in cancer treatment, particularly targeting the checkpoint proteins programmed cell death protein 1 (PD-1) and programmed cell death ligand 1 (PD-L1).^[6,7]^ While significant progress has been made in treating NSCLC with this approach, the effectiveness of PD-1 in managing advanced subcutaneous soft-tissue metastasis of lung adenocarcinoma remains uncertain. In this case study, we present a patient with recurrent lung cancer who developed subcutaneous soft-tissue metastasis and underwent combination treatment involving anti-PD-1 antibody and chemotherapy.

## 2. Case report

A 60-year-old man underwent radical resection of a 5.5 × 4.0 cm poorly differentiated adenocarcinoma located in the upper lobe of his right lung on March 22, 2020. Postoperatively, he was diagnosed with stage T2N1M0 (IIb) cancer, with no mutations detected in epidermal growth factor receptor, anaplastic lymphoma kinase, or ROS proto-oncogene 1. Following the surgery, he received 4 cycles of adjuvant chemotherapy consisting of pemetrexed and cisplatin, during which he experienced manageable gastrointestinal adverse effects. Regular follow-ups were conducted after chemotherapy. In June 2023, he noted the emergence of a painful mass on the left chest wall, which grew to 13.0 × 6.0 cm and exhibited signs of necrosis and bleeding. Computer tomography scans revealed metastatic tumors in the left axillary and adrenal regions, indicating disease progression. Immunohistochemical staining demonstrated negative PD-L1 expression. Consequently, the patient received treatment with tislelizumab (200 mg, a PD-1 inhibitor), pemetrexed (500 mg/m^2^), and carboplatin (area under the curve 5) chemotherapy on July 30, 2023.

Following the completion of the initial treatment cycle, the base of the large tumor, which was protruding from the skin surface, exhibited softening, with a reduction in bleeding and a significant decrease in pain; however, oral analgesics remained necessary. By the second treatment cycle, the tumor base had darkened and demonstrated signs of necrosis and desquamation. Bleeding ceased, pain was substantially alleviated, and the dosage of analgesics was reduced by half. Upon completion of the third cycle, the tumor fully detached, revealing a 10 cm ulcerated area. The patient received daily dressing changes and disinfection every other day, facilitating gradual healing. Pain management was effective, allowing the patient to discontinue the use of oral analgesics entirely. By the fourth cycle, the ulcerated skin area had decreased to approximately 6 cm, further reducing to 2 cm by the fifth cycle, and achieving complete healing by the sixth cycle.

**Figure 1.**
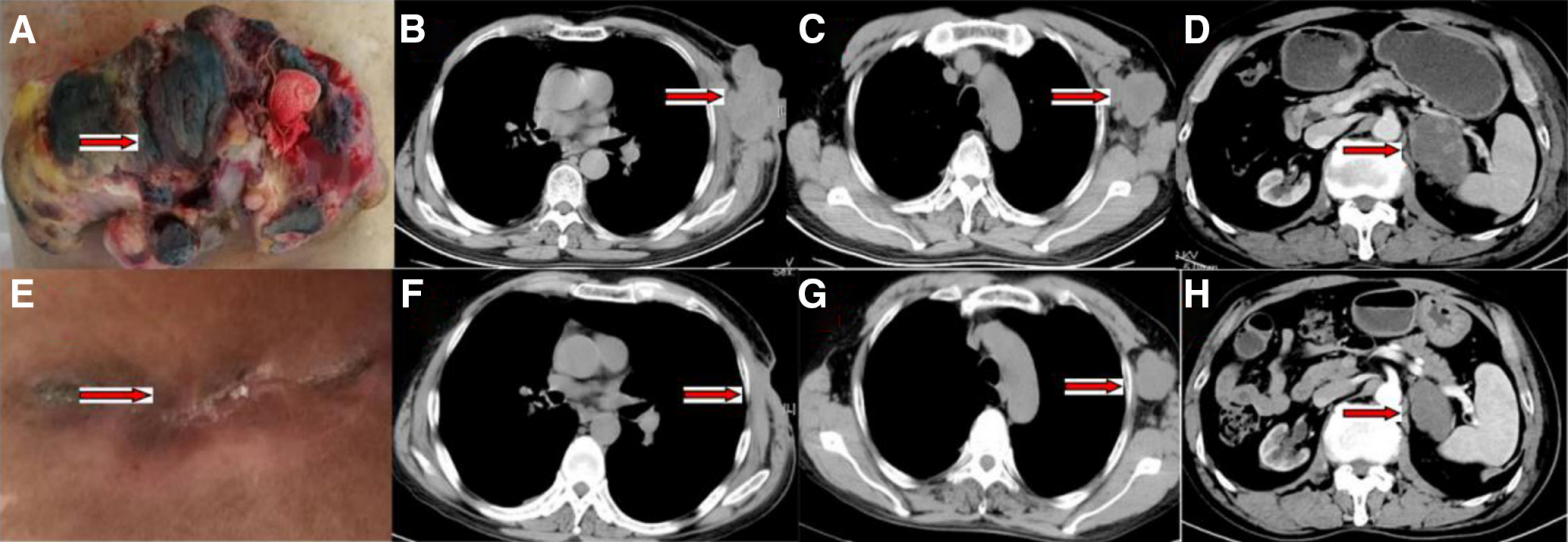
Initial CT scans: (A) Metastatic tumor measuring 13.0 × 6.0 cm located on the left thoracic wall, exhibiting necrotic hemorrhage on the surface; (B) subcutaneous soft-tissue metastasis of the left thoracic wall under CT; (C) left axillary lymph node metastatic tumor with a maximum diameter of 6.4 cm; (D) left adrenal metastatic tumor with a maximum diameter of 6.5 cm. After 6 cycles of medication: (E) Subcutaneous soft-tissue metastasis of the left thoracic wall has disappeared and the superficial skin began to heal; (F) subcutaneous soft-tissue metastasis of the left thoracic wall has disappeared under CT; (G) left axillary lymph node metastatic tumor with a maximum diameter of 3.7 cm; (H) left adrenal metastatic tumor with a maximum diameter of 5.1 cm. CT = computer tomography.

**Figure 2.**
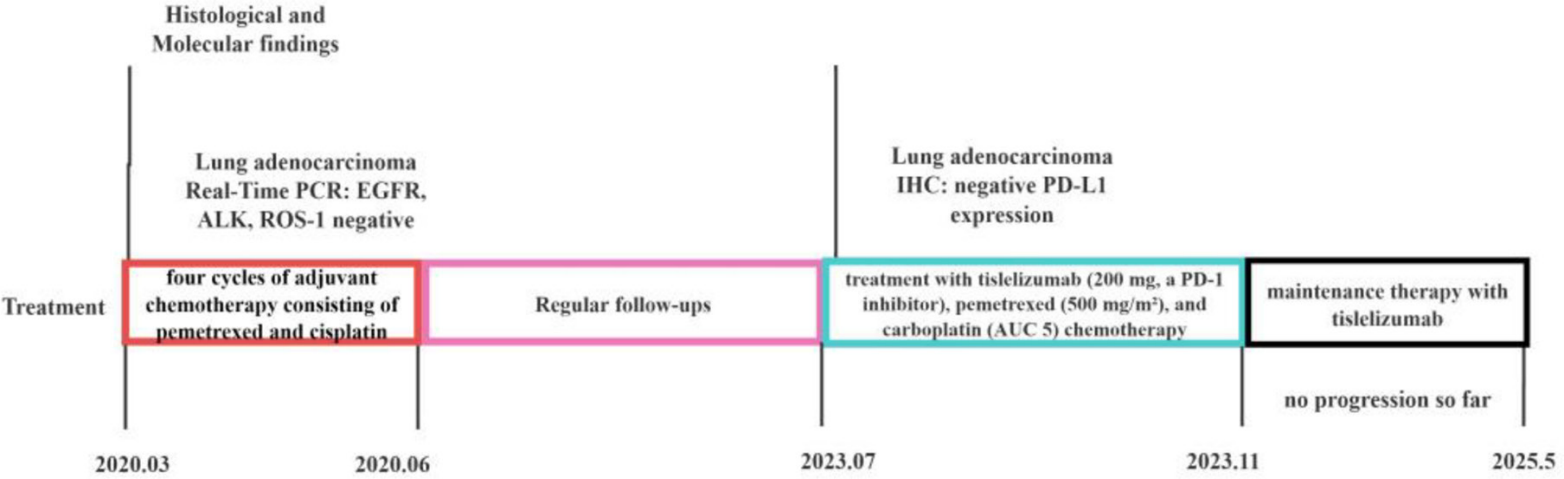
The patient’s treatment timeline. ALK = anaplastic lymphoma kinase, AUC = area under the curve, EGFR = epidermal growth factor receptor, IHC = immunohistochemistry, ROS-1 = ROS proto-oncogene 1, receptor tyrosine kinase, PCR = polymerase chain reaction, PD-L1 = programmed cell death ligand 1.

**Figure 3.**
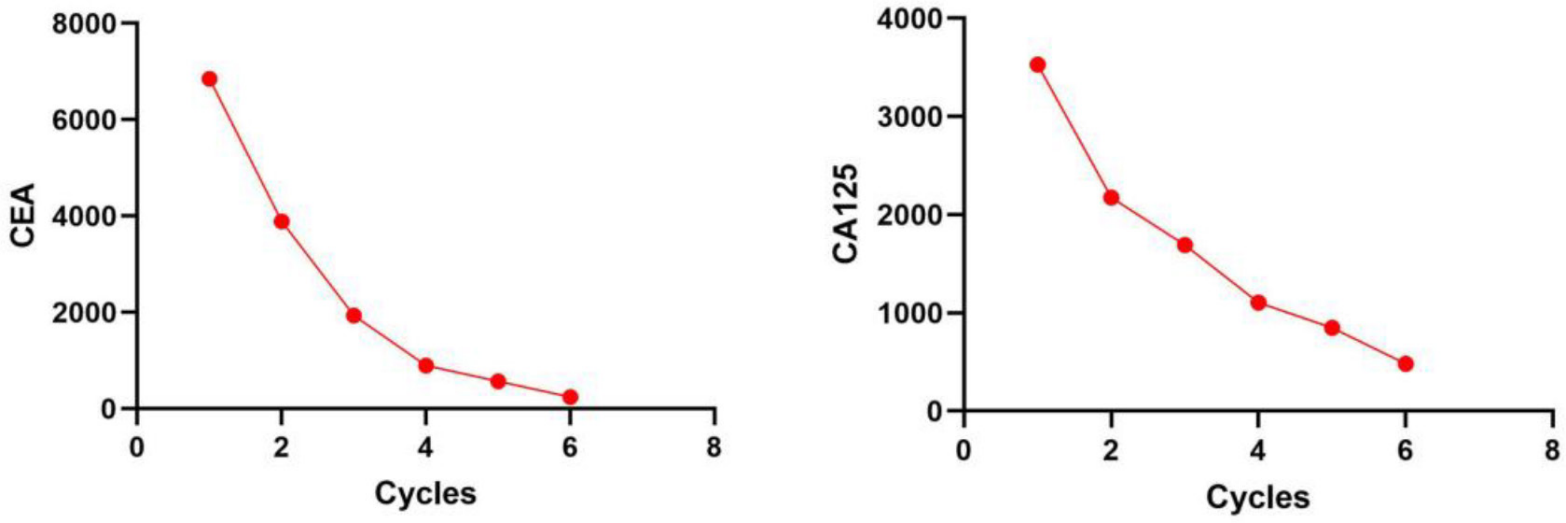
During the 6 treatment periods, the fluctuations in the patient’s tumor markers CEA and CA125. CA125 = carbohydrate antigen 125, CEA = carcinoembryonic antigen.

After 6 cycles of treatment, the results indicated the following: the large mass on the left chest wall exhibited signs of necrosis and shedding, with the skin over the lesion beginning to scab and heal; the left axillary lymph nodes and adrenal metastases demonstrated a reduction in size. The treatment was assessed as a partial response (Fig. 1). The patient’s treatment timeline is shown in Figure 2. During the 6 treatment periods, the fluctuations in the patient’s tumor markers carcinoembryonic antigen and carbohydrate antigen 125 are illustrated in Figure 3. The only adverse effects observed were thrombocytopenia (grade IV myelosuppression) and fatigue, both of which were effectively managed with symptomatic treatment. As of May 2025, the patient is still receiving maintenance therapy with tislelizumab.

## 3. Discussion

In recent years, immunotherapy has revolutionized the treatment of advanced NSCLC. Immune checkpoint inhibitors have become a cornerstone of systemic therapy, with combination regimens improving survival across multiple clinical settings. KEYNOTE-042, EMPOWER-Lung 1, and EMPOWER-Lung 3 confirmed that PD-1/PD-L1 inhibitors, alone or combined with chemotherapy, significantly improve overall survival and progression-free survival in advanced NSCLC, regardless of PD-L1 expression. For PD-L1-negative patients, KEYNOTE-189 and KEYNOTE-407 established pembrolizumab plus chemotherapy as a preferred first-line option, demonstrating consistent overall survival and objective response rate benefits.^[8–10]^

Subcutaneous soft-tissue metastasis is a rare and poor prognostic manifestation in advanced NSCLC, mostly originating from lung cancer and commonly involving the chest wall. It indicates high tumor burden and end-stage disease, with limited effective treatment options. Meanwhile, antibody-drug conjugates are rapidly developing in NSCLC. Early data from EVOKE-02^[11]^ and TROPION-Lung02 suggest that ADC plus immunotherapy ± chemotherapy exhibits promising efficacy and manageable safety in treatment-naïve metastatic NSCLC without actionable drivers, supporting further exploration of chemo-immunotherapy and ADC-immunotherapy combinations.

In this case, the patient with recurrent lung adenocarcinoma developed massive, symptomatic subcutaneous soft-tissue, axillary lymph node, and adrenal metastases after surgery and adjuvant chemotherapy. PD-L1 expression was negative. He was treated with an anti-PD-1 inhibitor plus pemetrexed and carboplatin. Remarkably, the large chest-wall metastatic lesion gradually necrosed, detached, and healed completely; lymph node and adrenal metastases shrank, and the overall efficacy was partial response. Treatment-related adverse events were grade IV thrombocytopenia and fatigue, which were well controlled with supportive care. The patient remained on maintenance anti-PD-1 therapy with durable disease control as of May 2025.

This case demonstrates that chemo-immunotherapy can achieve rapid and effective tumor regression in patients with bulky, symptomatic subcutaneous soft-tissue NSCLC metastases, even with negative PD-L1 expression. The combination therapy provides rapid symptom relief, high tumor response, and long-term disease control, supporting its role as a practical and effective treatment in this high unmet need population.

Future research should optimize combination regimens (chemo-immunotherapy, ADC-immunotherapy, radiotherapy-immunotherapy), establish multi-dimensional predictive biomarkers, develop novel agents to overcome resistance, and further validate the efficacy and safety of innovative combinations in advanced NSCLC.

## 4. Conclusion

In the field of advanced NSCLC, immunotherapy has achieved significant breakthroughs, reshaping the treatment landscape for this disease. The combination of immunotherapy and chemotherapy has benefited all driver gene-negative advanced NSCLC patients, resulting in increased survival rates and improved quality of life.

## Author contributions

**Conceptualization:** Qiang Zeng.

**Data curation:** Qiang Zeng, Yuan Zeng.

**Formal analysis:** Qiang Zeng.

**Methodology:** Bin Yang.

**Project administration:** Bin Yang.

**Resources:** Bin Yang.

**Validation:** Yang Hu.

**Visualization:** Yang Hu.

**Writing – original draft:** Yang Hu, Yuan Zeng.

**Writing – review & editing:** Yuan Zeng.
